# Partner Disclosure and Early CD4 Response among HIV-Infected Adults Initiating Antiretroviral Treatment in Nairobi Kenya

**DOI:** 10.1371/journal.pone.0163594

**Published:** 2016-10-06

**Authors:** T. Tony Trinh, Nelly Yatich, Richard Ngomoa, Christine J. McGrath, Barbra A. Richardson, Samah R. Sakr, Agnes Langat, Grace C. John-Stewart, Michael H. Chung

**Affiliations:** 1 Department of Global Health, University of Washington, Seattle, Washington, United States of America; 2 Department of Epidemiology, University of Washington, Seattle, Washington, United States of America; 3 Department of Medicine, University of Washington, Seattle, Washington, United States of America; 4 Department of Biostatistics, University of Washington, Seattle, Washington, United States of America; 5 Department of Pediatrics University of Washington, Seattle, Washington, United States of America; 6 Coptic Hospital, Nairobi, Kenya; 7 Department of Obstetrics and Gynecology, University of Texas Medical Branch, Galveston, Texas, United States of America; 8 US Center for Disease Control and Prevention, Nairobi, Kenya; Central University of Tamil Nadu, INDIA

## Abstract

**Background:**

Disclosure of HIV serostatus can have significant benefits for people living with HIV/AIDS. However, there is limited data on whether partner disclosure influences ART treatment response.

**Methods:**

We conducted a retrospective cohort study of newly diagnosed, ART-naïve HIV-infected adults (>18 years) who enrolled at the Coptic Hope Center in Nairobi, Kenya between January 1^st^ 2009 and July 1^st^ 2011 and initiated ART within 3 months. Analysis was restricted to adults who reported to have either disclosed or not disclosed their HIV status to their partner. Analysis of CD4 response at 6 and 12 months post-ART was stratified by age group.

**Results:**

Among 615 adults newly initiating ART with partner disclosure data and 12 month follow-up, mean age was 38 years and 52% were male; 76% reported that they had disclosed their HIV-status to their partner. Those who disclosed were significantly younger and more likely to be married/cohabitating than non-disclosers. At baseline, median CD4 counts were similar between disclosure groups. Among younger adults (< 38 years) those who disclosed had higher CD4 recovery than those who did not at 6 months post- ART (mean difference = 31, 95% CI 3 to 58 p = 0.03) but not at 12 months (mean difference = 17, 95% CI -19 to 52, p = 0.4). Among older adults (≥ 38years) there was no observed difference in CD4 recovery at 6 or 12 months between disclosure groups.

**Conclusion:**

Among younger adults, disclosure of HIV status to partners may be associated with CD4 recovery following ART.

## Introduction

Disclosure of HIV serostatus can have significant benefits for people living with HIV/AIDS. Disclosure has been associated with reduced anxiety, decreased rates of depression, and an increased sense of acceptance and strengthened relationships [1–3]. In the era of antiretroviral therapy (ART), disclosure has been linked to increase ART use [4], retention in care [5], and uptake of services such as prevention of mother-to-child transmission [6–7]. Disclosure to sexual partners has the added benefit of allowing informed choices that lead to risk behavior reduction to prevent HIV transmission [8–10]. In sub-Saharan Africa where approximately two-thirds of HIV incidence occurs in steady partnerships [11], disclosure remains an important priority in HIV testing and counseling programs.

HIV-infected persons who disclose their status may find additional support that motivates ART adherence. Those who disclose to their social network avail themselves of emotional and practical support with care appointments and treatment reminders. In steady partnerships, disclosure can provide an open home environment where pill taking need not be concealed. Additionally, knowledge that sustained viral suppression prevents transmission may provide additional motivation to adherence [12].

Despite these benefits, limited evidence exists on whether disclosure is associated with clinical outcomes such as ART treatment response. A small urban-based study reported a trend for higher 2-year post-ART CD4 counts among those who had disclosed [5]. It is well established that immune response is associated with baseline CD4 and age [13–14], but there have been no published studies to our knowledge evaluating the association between partner disclosure and immune recovery following ART.

In this study, we performed an age-stratified comparison of CD4 response post-ART among HIV-infected individuals who reported disclosing their HIV status to their steady partner (disclosers) versus those who did not disclose (non-disclosers).

## Methods

We conducted a retrospective cohort study using data from the Coptic Hope Center for Infectious Disease in Nairobi, Kenya. The Hope Center is funded by the President’s Emergency Plan for AIDS Relief (PEPFAR), administered by the Coptic Orthodox Mission with support from the University of Washington, and has enrolled over 20,000 HIV-infected clients and provided free ART to over 15,000 clients since 2004.

Adults (>18 years) enrolling in care between January 1^st^ 2009 and July 1^st^ 2011 were eligible for analysis. We included adults who were newly diagnosed (first HIV confirmatory test within 3 months), ART-naïve, had a steady partner, and initiated ART within 3 months of enrollment. ART used at the Hope Center was in accordance with WHO guidelines. At enrollment, all clients were interviewed using standardized forms to capture demographic information (age, gender, educational level and employment status).

Partnership and disclosure status were ascertained during pre-ART counseling sessions and recorded on paper forms that were scanned into a database using TeleForm software (Autonomy, Cardiff, Vista, California, USA). Using a standardized questionnaire (S1 File), the client was asked, “how many spouse(s) or steady partner(s) do you have?”. Any client who reported no partner was excluded from analysis. Regarding disclosure, the client was queried “have you revealed your status to your spouse(s) or steady partner(s)”. The answer options were “all” “some” “none” or “has no partner / spouse”. A client was determined to have disclosed to their partner, if they had answered “all” or “some” to the “spouse(s) or steady partner(s)” category of the disclosure question. Partner non-disclosure was determined if the client had answered “none”. Clients with unrecorded answers to the partner or disclosure questions and clients with discordant answers (e.g. > 0 to the partner question, and “has no partner” to the disclosure question) were excluded.

As part of routine care, clients on treatment were evaluated every 3 months. CD4 measurements [BD FACSCalibur, San Jose, California] were taken before ART initiation, 6 and 12 months after initiation within a 60-day time window. The immune recovery analysis included clients with CD4 measurements at both 6 and 12 months post-ART. Clients missing both or either 6 or 12 month post-ART CD4 measurements were excluded.

Chi square tests were used to measure differences between categorical baseline characteristics. A two-sample t-test was used to measure differences between normally distributed continuous baseline variables (i.e., age and time to ART initiation). Wilcoxon-Mann-Whitney test was used to determine differences between CD4 counts at baseline, 6 and 12 months post-ART. CD4 response analysis was stratified by age, categorized as a binary variable using the mean age as the cut off. “Younger adults” comprised those below the mean age. “Older adults” were those including the mean age and older. Multivariable linear regression was used to determine differences in CD4 response from baseline to 6 months and baseline to 12 months, after adjusting for marital status, gender, and baseline CD4. All analyses were performed using Stata/SE 11.2 software (StataCorp). All data was de-identified prior to analysis.

## Results

Between January 2009 and July 2011, 3414 adults were enrolled at the Hope Center. Of these, 246 (7%) were previously diagnosed, 286 (8%) were ART-experienced, 1262 (37%) did not initiate ART within 3 months, 702 (21%) did not report a spouse or steady partner, 100 (3%) had incomplete disclosure or partner data, and 203 (6%) were missing 6 or 12-month CD4 measurements. The analysis ultimately included 615 (18%) adult clients who were newly diagnosed, initiated ART within 3 months, and had CD4 count data at 6 and 12 months post-ART.

Among the analysis cohort, mean age was 38.3 years [95% Confidence Interval (CI) 37.6–39.0) and 52% were male. A large majority were married/cohabitating (90%), had a secondary education or higher (73%), and were employed (78%) (Table 1). Disclosure was reported in 76% of the cohort. Disclosers were significantly younger (37.8 versus 40.0 years, p <0.02) and more likely to be married/cohabitating (93% versus 80%, p<0.001) than non-disclosers. There was no difference in baseline median CD4 count between disclosers (125 cells/μL) and non-disclosers (109 cells/ μL; p = 0.15).

**Table 1:** Characteristics of adults with steady partners (n = 615) initiating ART, but disclosure status at enrollment.

Characteristic	Total	Disclosed	Not Disclosed	p value
	615	468	147	
Female, n(%)	296 (48%)	227 (49%)	69 (47%)	0.74
Male, n(%)	319 (52%)	241 (51%)	78 (53%)	
Married/cohabitating, n (%)	551 (90%)	434 (93%)	117 (80%)	**<0.001**
Secondary Education or higher, n (%)	449 (73%)	347 (74%)	102 (69%)	0.26
Unemployed, n (%)	136 (22%)	111 (24%)	25 (17%)	0.09
Age, mean years (95% CI)	38.3 (37.6–39.0)	37.8 (37.0–38.6)	40.0 (38.3–41.3)	**0.02**
Enrollment to ART initiation, median days (IQR)	30.0 (28.8–31.2)	30.2 (28.8–31.6)	29.3(26.7–31.8)	0.53
Baseline CD4 measurement, median CD4 cells/uL (IQR)	123 (50–209)	125 (51–214)	109 (48–173)	0.15

Among younger adults (< 38 years), disclosers had a higher CD4 recovery at 6 months post-ART than non-disclosers after adjusting for gender, baseline CD4 count (mean difference = 31, 95% CI 3 to 58 p = 0.03) but not at 12 months (mean difference = 17, 95% CI -19 to 52, p = 0.4) (Table 2). Among older adults (≥ 38 years) there was no observed difference in CD4 recovery at 6 or 12 months between disclosure groups.

**Table 2:** Average difference in CD4 response* between disclosers versus non-disclosers following ART initiation stratified by age.

	Age <38 (314)		Age ≥ 38 (301)	
Months post-ART	Mean difference (95% CI)	p value	Mean difference (95% CI)	p value
6	31 (2.7 to 58)	**0.03**	15 (-12 to 41)	0.3
12	17 (-19 to 52)	0.4	6 (-22 to 33)	0.7

*adjusted for marital status, baseline CD4 count, gender, time to measurement.

## Discussion

In our retrospective cohort of new enrollees initiating ART we found a majority (76%) had disclosed their HIV serostatus to their partners. At baseline, disclosers were younger and more likely to be married or cohabitating with their partners compared to non-disclosers. Among younger adults, disclosers had a significantly higher average CD4 response compared to non-disclosers at 6 months after ART initiation.

The prevalence of partner disclosure (76%) in our study is similar to that observed in other studies in sub-Saharan Africa. Studies from a variety of sites in South Africa found the prevalence of disclosure was approximately 80% [15–16]. Not surprisingly, disclosure was more common among married partners in our study. HIV-positive individuals have reported a greater sense of responsibility to disclose to partners with whom there was a shared emotional relationship [17]. Similar to our study, many other studies have observed a lower prevalence of disclosure among older clients. Observational studies in both resource rich and limited settings have shown that HIV-infected older adults are less likely to disclose to partners, friends and family due in part to an increased sense of stigma [15,17–22].

It is also well established that older age is associated with lower CD4 response following ART. Thus, it is not surprising that among older adults in our study, we found no differences in CD4 response between disclosure groups at 6 or 12 months post ART. Viard and colleagues showed that older age was associated with both a lower absolute CD4 cell gain and a longer time to maximum response independent of baseline CD4 count and other factors effecting CD4 response [14]. Given the blunted CD4 response among older adults, any association between disclosure and CD4 response in this group would likely have required a much larger sample size and longer follow up period than our study was designed for.

However, among younger adults, we found that disclosers had a significantly higher CD4 response at 6 months of ART, than non-disclosers. Difference in early treatment response is likely associated with a difference in ART-adherence patterns. In a cross-sectional study in rural Zambia, partner disclosure and knowledge of partner status was associated with improved adherence [23]. Similarly, in a US-based cross-sectional study assessing ART adherence tracked by bottle cap devices, and self-reported disclosure, there was higher adherence among those with HIV disclosure [24]. Conversely, non-disclosure limits the potential for support persons to assist with treatment reminders and limits the ability to take ART openly. One study noted disclosure-related reasons for missing ART doses as “I didn’t want others to notice me taking medication” and “I was with people who didn’t know I was HIV positive” [24]. As it is well known that suboptimal adherence predisposes to suboptimal treatment response [25], our finding provides important complementary evidence regarding the clinical benefits of disclosure especially for those starting ART.

At 12 months post-ART, CD4 counts did not differ significantly between disclosure groups. One potential reason for this is that our ascertainment of disclosure status was only at baseline. Studies have shown that disclosure is a dynamic process that occurs over time. In a large cohort in Tanzania, prevalence of disclosure was 22% within 2 months to 40% nearly 4 years after diagnosis [26]. It is likely that a proportion of non-disclosers in our study disclosed after enrollment, thus attenuating any CD4 differences over time.

There are additional limitations to our study. This was a single-site study in an urban setting in Kenya using retrospective data. We did not have data on adherence and thus could not analyze its potential role as a mediator between disclosure and CD4 response. We did not control for active co-morbid diseases that could have affected CD4 measurements independent of ART. We restricted our disclosure definition to steady partners and excluded disclosure to friends, family, or other social support persons. There is growing evidence that disclosure to a partner/spouse is a distinct process separate from disclosure to friends and family [27] and that those who choose to disclose between family and friends may differ from those who choose to disclose to partners exclusively [18]. Any association between clinical outcome and disclosure may be more linked to disclosure recipients identified as social supporters independent of their identities as partners. Additionally, we restricted analysis to retained clients in order to ascertain CD4 data. This excluded those with significant difficulties engaging in care who may be the most affected by stigma and most reluctant to disclose.

## Conclusion

In conclusion, our study is the first to analyze the association between early CD4 response and partner disclosure among ART-naïve patients initiating treatment. Our results suggest that partner disclosure may be associated with early CD4 recovery following ART initiation among younger adults. More research is needed to better understand the dynamics of disclosure and the impact/role of disclosure on clinical outcomes in HIV-infected patients initiating ART.

## Supporting Information

S1 File Hope Clinic Counselor Screening Form. (PDF)
