# Supplementary material for: Partner Disclosure and Early CD4 Response among HIV-Infected Adults Initiating Antiretroviral Treatment in Nairobi Kenya
Source: PLoS One. 2016 Oct 6;11(10):e0163594. doi: 10.1371/journal.pone.0163594 (PMC5053490; doi:10.1371/journal.pone.0163594)
Supplement: S1 File — (PDF) [file pone.0163594.s001.pdf]

# HOPE CLINIC

## COUNSELOR SCREENING



**HOPE ID Number**

**Site Code** | **Today's date (DD.MM.YYYY)** | **Interviewer number**

☐☐☐☐☐ | ☐☐ / ☐☐ / ☐☐☐☐ | ☐☐☐

1. How many children do you have or care for? ☐☐ *If = 0 go to 2*

1a. Describe HIV test results and HIV care for each child.

| | Test Result | Receiving HIV care? | Receiving HAART? | Recieving HAART and/or HIV care at Hope Clinic? |
|---|---|---|---|---|
| 1 | ☐ Positive ☐ Negative ☐ Unknown ☐ Not tested | ☐ Yes ☐ Unknown ☐ No ☐ N/A | ☐ Yes ☐ Unknown ☐ No ☐ N/A | ☐ Yes ☐ Unknown ☐ No ☐ N/A |
| 2 | ☐ Positive ☐ Negative ☐ Unknown ☐ Not tested | ☐ Yes ☐ Unknown ☐ No ☐ N/A | ☐ Yes ☐ Unknown ☐ No ☐ N/A | ☐ Yes ☐ Unknown ☐ No ☐ N/A |
| 3 | ☐ Positive ☐ Negative ☐ Unknown ☐ Not tested | ☐ Yes ☐ Unknown ☐ No ☐ N/A | ☐ Yes ☐ Unknown ☐ No ☐ N/A | ☐ Yes ☐ Unknown ☐ No ☐ N/A |
| 4 | ☐ Positive ☐ Negative ☐ Unknown ☐ not tested | ☐ Yes ☐ Unknown ☐ No ☐ N/A | ☐ Yes ☐ Unknown ☐ No ☐ N/A | ☐ Yes ☐ Unknown ☐ No ☐ N/A |

2. Have you revealed your serostatus to:

a. Spouse(s) or steady partner(s) ○ All ○ Some ○ None ○ Has no spouse

b. Casual or non-casual partner(s) ○ All ○ Some ○ None ○ Has no partner

c. Parents ○ All ○ Some ○ None ○ Has no parents

d. Siblings ○ All ○ Some ○ None ○ Has no siblings

e. Children ○ All ○ Some ○ None ○ Has no children

f. Friends ○ All ○ Some ○ None ○ Has no friends

g. Others ○ All ○ Some ○ No one else ○ Specify ________

3. Who forms your closest social support (tick one)

☐ Spouse/steady partner ☐ Sibling

☐ Mother ☐ Other (specify) ________

☐ Father ☐ No social supporter

☐ Friend

3a. Have you informed this person of your serostatus?

☐ Yes ☐ No ☐ N/A

4. Have you ever had sex? ☐ Yes ☐ No *(If YES, go to 5, If NO go to 13)*

5. How many spouse(s) or steady partner(s) do you have? ☐☐ *(If = 0 go to 6)*

5a. Describe HIV test results and HIV care for each spouse(s) or steady partner(s)

| | Relationship | Test Result | Receiving HIV care? | Receiving HAART? | Recieving HAART and/or HIV care at Hope Clinic? |
|---|---|---|---|---|---|
| 1 | ☐ Spouse<br>☐ Steady partner | ☐ Positive<br>☐ Negative<br>☐ Unknown<br>☐ not tested | ☐ Yes<br>☐ No<br>☐ Unknown<br>☐ N/A | ☐ Yes<br>☐ No<br>☐ Unknown<br>☐ N/A | ☐ Yes<br>☐ No<br>☐ Unknown<br>☐ N/A |
| 2 | ☐ Spouse<br>☐ Steady partner | ☐ Positive<br>☐ Negative<br>☐ Unknown<br>☐ Not tested | ☐ Yes<br>☐ No<br>☐ Unknown<br>☐ N/A | ☐ Yes<br>☐ No<br>☐ Unknown<br>☐ N/A | ☐ Yes<br>☐ No<br>☐ Unknown<br>☐ N/A |
| 3 | ☐ Spouse<br>☐ Steady partner | ☐ Positive<br>☐ Negative<br>☐ Unknown<br>☐ Not tested | ☐ Yes<br>☐ No<br>☐ Unknown<br>☐ N/A | ☐ Yes<br>☐ No<br>☐ Unknown<br>☐ N/A | ☐ Yes<br>☐ No<br>☐ Unknown<br>☐ N/A |
| 4 | ☐ Spouse<br>☐ Steady partner | ☐ Positive<br>☐ Negative<br>☐ Unknown<br>☐ not tested | ☐ Yes<br>☐ No<br>☐ Unknown<br>☐ N/A | ☐ Yes<br>☐ No<br>☐ Unknown<br>☐ N/A | ☐ Yes<br>☐ No<br>☐ Unknown<br>☐ N/A |
| 5 | ☐ Spouse<br>☐ Steady partner | ☐ Positive<br>☐ Negative<br>☐ Unknown<br>☐ Not tested | ☐ Yes<br>☐ No<br>☐ Unknown<br>☐ N/A | ☐ Yes<br>☐ No<br>☐ Unknown<br>☐ N/A | ☐ Yes<br>☐ No<br>☐ Unknown<br>☐ N/A |
| 6 | ☐ Spouse<br>☐ Steady partner | ☐ Positive<br>☐ Negative<br>☐ Unknown<br>☐ Not tested | ☐ Yes<br>☐ No<br>☐ Unknown<br>☐ N/A | ☐ Yes<br>☐ No<br>☐ Unknown<br>☐ N/A | ☐ Yes<br>☐ No<br>☐ Unknown<br>☐ N/A |

6. How many diffrerent casual or non-steady partner(s) did you have in the past year? ☐☐

7. In the past year, how many different sexual partners have you had, including your spouse(s) and steady partner(s)? ☐☐☐

8. During your lifetime have you had sex with

☐ Men Only ☐ Women Only ☐ Both ☐ None ☐ Refused to answer

9. Describe your condom use in the past 12 months todate:-

a.Spouse(s) or steady partiners *(Tick one)*

☐ Never ☐ Sometimes ☐ Always ☐ No sex in past 12 months ☐ No spouse/steady partner

b.Non- steady partiners. *(Tick one)*

☐ Never ☐ Sometimes ☐ Always ☐ No sex in past 12 months ☐ No Non-steady partner

10. Did you use a condom during your last sexual encounter

☐ No ☐ Yes ☐ Refused to answer

11. Are you able to talk about using condoms with your spouse(s) or steady partner(s)?

☐ No ☐ Yes ☐ Refused to answer ☐ No spouse/steady partner ☐ Dont know

11a. Are you able to say NO to sex if your spouse or steady partner will not use a condom?

☐ No ☐ Yes ☐ No spouse/steady partner ☐ Refused to answer ☐ Don't know

12. Are you able to talk about condoms with your casual or non-steady partner(s)?

☐ Yes ☐ No ☐ No non-steady partner(s) ☐ Refused to answer ☐ Don't know

12a. Are you able to say NO to sex if your casual or non-steady partner(s) will not use a condom?

☐ No ☐ Yes ☐ No non-steady partner(s) ☐ Refused to answer ☐ Don't know

13. Are you Circumcised ? ***(Ask Male client only)***

☐ Yes ☐ No ☐ Refused to answer ☐ Female client

14. Do you feel neglected by anyone *(tick all that apply)*

☐ Family ☐ Friends ☐ Health care workers ☐ None ☐ Others (specify) ____________

15. Counselor, does the client need a treatment supporter? ☐ Yes ☐ No *(If yes go to 15a, if no go 16)*

15a. If YES why? specify (Tick all that apply)

☐ Client requests treatment supporter

☐ Physically disabled

☐ Mentally disabled

☐ Other (specify) ____________

16. Counselor is this client recommended for homecare? ☐ Yes ☐ No

16a. If YES, specify why? (Tick all that apply)

- ☐ Physically or mentally disabled adult
- ☐ Adult dependent on care-giver
- ☐ Client requests a home visit
- ☐ Disclosure (patient would like help disclosing status to family members)
- ☐ Family testing (client would like other family members to be tested)
- ☐ Other (specify) ________

17. Have you explained/discussed or checked the following with the Client?

| Checklist | Tick (if Yes) | Tick (if No) |
|---|---|---|
| 1. Overview of HOPE Center Program and Services | ○ | ○ |
| 2. Importance of commitment to the program | ○ | ○ |
| 3. Policy for adherence and clinic attendance | ○ | ○ |
| 4. Patients enrollment status in other programs or facilities | ○ | ○ |
| 5. Patients long term goals for health management at our program | ○ | ○ |

18. Did the patient pick condoms? ☐ Yes ☐ No

Notes / Assessment

*For the data use only (tick after scanning the form)*

*Scanned* ☐ *Date* ___ / ___ / _________ *Name of data person* ________
